# New model for predicting the development of pancreatic pseudocyst secondary to acute pancreatitis

**DOI:** 10.1097/MD.0000000000036102

**Published:** 2023-11-24

**Authors:** Shanbing Hou, Senlin Wang, Yuetong You, Lanlan Yang, Ming Dou, Ying Zhang

**Affiliations:** a Department of Emergency, The First Affiliated Hospital of USTC, Division of Life Sciences and Medicine, University of Science and Technology of China, Hefei, Anhui, China; b Graduate College of Wannan Medical College, Wuhu, Anhui, China.

**Keywords:** decision-making, risk prediction, severe pancreatitis

## Abstract

Pancreatic pseudocyst (PPC) increases the risk of a poor prognosis in in patients with acute pancreatitis (AP). Currently, an efficient tool is not available for predicting the risk of PPC in patients with AP. Therefore, this research aimed to explore the risk factors associated with PPC secondary to AP and to develop a model based on clinical information for predicting PPC secondary to AP. This study included 400 patients with acute pancreatitis and pancreatic pseudocyst secondary to acute pancreatitis admitted to the emergency department and gastroenterology department of The First Affiliated Hospital of the University of Science and Technology of China from January 2019 to June 2022. Participants were divided into no PPCs (321 cases) and PPCs (79 cases). Independent factors of PPC secondary to AP were analyzed using univariate and multivariate logistic regression. The nomogram model was constructed based on multivariate logistic regression analyses, which included all risk factors, and evaluated using R. We enrolled 400 eligible patients and allocated 280 and 120 to the training and test sets, respectively. Clinical features, including severe pancreatitis history [odds ratio (OR) = 4.757; 95% confidence interval (CI): 1.758–12.871], diabetes mellitus (OR = 6.919; 95% CI: 2.084–22.967), history of biliary surgery (OR = 9.232; 95% CI: 3.022–28.203), hemoglobin (OR = 0.974; 95% CI: 0.955–0.994), albumin (OR = 0.888; 95% CI: 0.825–0.957), and body mass index (OR = 0.851; 95% CI: 0.753–0.962), were significantly associated with the incidence of PPC after AP in the training sets. Additionally, the individualized nomogram demonstrated good discrimination in the training and validation samples with good calibration, The area under the curve and 95% CI of the nomogram were 0.883 (0.839–0.927) in the training dataset and 0.839 (0.752–0.925) in the validation set. We developed a nomogram model of PPC secondary to AP using R Studio. This model has a good predictive value for PPC in patients with AP and can help improve clinical decision-making.

**Key Points**

- Strengths and limitations of this study: This research aimed to explore the risk factors associated with PPC secondary to AP and develop a model based on clinical information for predicting PPC secondary to AP.
- This is a single-center retrospective study; hence, the sample source and size are insufficient, and the effect of regional characteristics on the disease cannot be excluded.

## 1. Introduction

Acute pancreatitis (AP) is a common inflammatory disease of the digestive system caused by acinar cells. Inflammatory cells in AP activate the release of inflammatory factors, leading to proinflammatory and anti-inflammatory state disorders. Therefore, the probability of AP progressing to severe pancreatitis (SAP) is high. Moreover, AP progression to critical illness is often accompanied by different degrees of persistent multi-organ function damage or failure and body complications. Thus, the case-fatality rate is relatively high.^[1]^ Most patients with pancreatitis recover quickly after conservative clinical treatment, but few patients may progress to secondary AP-related complications.

Pancreatic pseudocyst (PPC) is a common clinical complication in patients with AP and SAP. With the incidence of pancreatitis increasing yearly, the probability of secondary PPC is gradually increasing. A previous study discovered that the probability of a PPC secondary to AP ranges from approximately 2% to 18.5%.^[2]^ PPCs are pathologically and anatomically peripancreatic effusions rich in higher amylase and wrapped in a fibrous granulation tissue wall.^[3]^ Furthermore, PPCs aggravate the patients’ conditions and increase mortality. Studies have shown that large PPCs will compress multiple organs in patients, possibly causing various clinical complications, such as infection and bleeding, aggravating the patients’ conditions and resulting in death. From the perspective of public medical resource allocation, PPCs affect the disease prognoses of patients, leading to repeated hospitalization. As a result, this increases the average hospitalization days of patients and the expenditure burdens of medical insurance funds. Therefore, the main task of continuous treatment in the recovery period of AP is to identify the influencing factors leading to PPC secondary to AP and to conduct high-quality screening of the target population prone to complicated PPC, intervention, and strict follow-up. The definition of nomogram is a graphical analysis tool used to calculate the relationship between multiple variables and a specific outcome. It is typically composed of a set of straight lines, coordinate axes, and scales, and provides a simple yet reliable method for predicting patient disease risk or incidence rate. Therefore, this study aimed to develop efficient risk prediction tools for secondary PPCs in patients with AP, improve the efficiency of pre-onset screening for PPCs, and reduce the incidence of PPCs. ^[4]^

## 2. Methods

### 2.1. Study participants

This single-center, retrospective, observational research was approved by the medical institutional ethics committee at The First Affiliated Hospital of the University of Science and Technology of China (Anhui Provincial Hospital) (approval number: 2022-RE-404). This study is retrospective nature, so the requirement for individual informed consent was waived by the ethics committee. The data were maintained with confidentiality to protect privacy of the participants. All the procedures were performed under principles of the local law and the Declaration of Helsinki. The study collected examination results and all relevant medical records of patients admitted to our hospital between January 2019 and June 2022. This study included 321 patients with AP and 79 patients with PPC secondary to AP. Inclusion criteria were as follows: patients with a discharge diagnosis of acute or severe pancreatitis and complete medical records; patients with PPC at discharge who can be traced back to initial AP medical records; and patients who underwent abdominal ultrasonography and computed tomography. Exclusion criteria were as follows: Patients with incomplete medical records or medical records that are not rigorously written; patients with chronic pancreatitis and acute attacks of chronic pancreatitis; patients who abandoned treatment after disease diagnosis; patients who died during their hospital stay; and patients with drugs, immunity, and other pancreatic damage.

### 2.2. Data collection and diagnostic criteria

The patients data for this study were collected retrospectively, including general demographic data, sex, age, history of SAP, diabetes mellitus, body mass index (BMI), history of smoking, history of drinking, history of biliary surgery, white blood cells (WBCs), percentage of neutrophil, neutrophil absolute value, red blood cells (RBCs), hemoglobin (HGB), platelet, alanine transferase (ALT), aspartate transferase (AST), alkaline phosphatase (ALP), total bilirubin (TBIL), direct bilirubin, indirect bilirubin (IBIL), TP, albumin (ALB), calcium, creatinine (CREA), blood urea nitrogen, uric acid, and blood glucose. Diagnostic criteria for AP were based on the guidelines for diagnosing and treating AP, according to the definition in the 2012 revision of the Atlanta classification.^[5]^ Furthermore, AP was diagnosed based on at least 2 of the 3 following criteria: AP diagnosis made based on clinical criteria; lipase or serum amylase activity at least 3 times greater than the upper limit of normal; and imaging corresponding to the imaging characteristics of AP. Diagnostic criteria for PPC were as follows^[6]^: pancreatitis history; identification of pancreatic cysts by ultrasound or imaging adjuvant examination 4 weeks after regular pancreatitis treatment; and cysts able to be observed dynamically, excluding pancreatic cystic tumors.

### 2.3. Statistical analysis

Data were analyzed and counted using SPSS 22.0 and R Studio (R3.6.1) software. The data was divided into training and validation sets in a 7:3 ratio using the random number table method. All measurement data were tested for normal distribution; normally distributed measurements are expressed as means ± standard deviations, and measurements that did not meet the normal distribution are represented by M (P25, P75). Between-group contrasts of measurement data were analyzed using an independent sample *t* test or the Mann–Whitney *U* test. Categorical variables are each represented by the number of examples (n) and percentage (%). Between-group comparisons were tested using the Pearson Chi-square test. Significant influencing factors in the univariate analysis were included in the multivariate logistic regression models. Influencing factors selected from the multivariate logistic regression analysis were included in the R Studio (R3.6.1) software. A nomogram prediction model for the risk of secondary PPC in patients with AP was drawn using the “rms” package.^[7]^ The nomogram model was interpreted based on the following method: Read the target value in the top point coordinate axis at the top of the corresponding target value in the coordinate of each influencing factor on the nomogram. The specific score values in the point coordinate axis of the corresponding target value of all influencing factors are summed up to obtain the total score value “Total Point”. Find the corresponding value in the total score (Total Point) axis, and intersect the vertical line and the “risk value” axis to obtain the risk probability of secondary PPC in patients with AP. The study group referred to the declaration of the TRIPOD prediction model to prevent overfitting after establishing the nomogram model.^[8]^ The established nomogram model was internally verified using Bootstrap autonomous sampling 1000 times. The predictive power of the validation model was detected using the internal validation model of the area under the curve (AUC) of the receiver operating characteristics curve. The prediction consistency and calibration of this nomogram were evaluated using the Hosmer–Lemeshow goodness of fit test and the model’s calibration curve.

## 3. Results

### 3.1. Baseline characteristics of patients

Table 1 presents the baseline data of patients in the training and validation sets. The incidence of PPC was comparable between the training and validation sets (19.28% vs 20.83%). However, both data groups were not statistically significant regarding the other comorbidities, such as SAP history, BMI, and PLB.

**Table 1:** Characteristics of acute pancreatitis patients in the training and validation sets.

	Training	Validation	*P* value
Pancreatic pseudocyst experience			.409
Non-pancreatic pseudocyst	226 (80.7)	95 (79.2)	
Pancreatic pseudocyst	54 (19.3)	25 (20.8)	
History of severe pancreatitis			
No	238 (85.0)	107 (89.2)	.171
Yes	42 (15.0)	13 (10.8)	
Sex			.535
Male	180 (64.3)	77 (64.2)	
Female	100 (35.7)	43 (35.8)	
Hypertension			.381
No	217 (77.5)	91 (75.8)	
Yes	63 (22.5)	29 (24.2)	
Diabetes mellitus			.451
No	250 (89.3)	106 (88.3)	
Yes	30 (10.7)	14 (11.7)	
History of biliary surgery			.086
No	251 (89.6)	101 (84.2)	
Yes	29 (10.4)	19 (15.8)	
History of smoking			.426
Yes	66 (23.6)	30 (25.0)	
No	214 (76.4)	90 (75.0)	
History of drinking			.475
Yes	63 (22.5)	28 (23.3)	
No	217 (77.5)	92 (76.7)	
Abdominal cavity or pelvic effusion			.127
No	195 (69.6)	91 (75.8)	
Yes	85 (30.3)	29 (24.2)	
HGB	132.51 ± 22.84	136.83 ± 18.33	.067
ALB	39.05 ± 5.87	40.65 ± 6.15	.014
GLO	28.18 ± 5.52	27.71 ± 5.34	.430
UA	310.15 ± 119,75	309.04 ± 116.13	.932
Age	46.00 (33.00, 46.00)	49.00 (33.00, 60.00)	.545
BMI	24.22 (22.41, 27.06)	23.97 (22.11, 25.88)	.831
WBC	8.95 (6.05, 12.93)	9.17 (5.99, 9.17)	.969
Percentage of neutrophils	76.40 (63.30, 84.40)	73.65 (62.68, 82.35)	.624
Neutrophil absolute value	6.80 (4.04, 11.10)	6.92 (3.79, 10.26)	.787
RBC	4.34 (3.85, 4.83)	4.37 (4.07, 4.69)	.325
PLT	202.00 (151.00, 264.00)	212.00 (168.75, 257.50)	.241
ALT	32.00 (16.00, 71.00)	33.10 (19.00, 81.00)	.247
AST	25.00 (17.00, 47.40)	28.00 (19.00, 53.50)	.140
ALP	89.00 (67.00, 126.00)	95.00 (75.25, 138.50)	.056
TBIL	18.10 (13.00, 25.80)	17.55 (12.13, 25.90)	.953
DBIL	6.80 (4.70, 11.70)	6.60 (4.12, 10.30)	.977
IBIL	10.50 (7.10, 15.00)	10.34 (6.60, 15.85)	.630
TP	66.40 (61.40, 71.90)	65.95 (61.40, 70.83)	.482
CA	2.19 (2.06, 2.31)	2.22 (2.09, 2.29)	.261
CREA	60.30 (47.00, 73.00)	61.50 (50.25, 71.00)	.994
BUN	4.26 (3.20, 5.40)	4.09 (3.10, 5.33)	.645
GLU	6.34 (5.18, 8.13)	6.12 (5.13, 8.07)	.529

ALB = albumin, ALP = alkaline phosphatase, ALT = alanine transferase, AST = aspartate transferase, BMI = body mass index, BUN = blood urea nitrogen, CA = blood calcium, CREA = creatinine, DBIL = direct bilirubin, GLO = glutamate oxidase, GLU = blood glucose, HGB = Hemoglobin, IBIL = indirect bilirubin, PLT = platelet, RBC = red blood cell, TBIL = total bilirubin, TP = total protein, UA = uric acid, WBC = white blood cell.

### 3.2. Univariate analysis of the risk of secondary PPC in patients with AP

In the training set of 280 patients, with secondary PPCs in 54 patients, the secondary incidence rate was approximately 19.29%. There were 180 male patients (64.3%) and 100 female patients (35.7%), with a mean age of 47.35 ± 15.50 years. Patients with secondary PPCs were aged 49.56 ± 12.84 years, while those without secondary PPCs were aged 46.82 ± 16.06 years. Univariate analysis revealed 19 significant variables, including diabetes mellitus, history of biliary surgery, abdominal effusion, RBC, HGB, IBIL, TP, ALB, BMI, WBC, percentage of neutrophils, neutrophil absolute value, ALT, AST, ALP, TBIL, CA, and CREA (*P* < .05; Table 2).

**Table 2:** Characteristics of acute pancreatitis patients in pancreatic pseudocyst set and non-pancreatic pseudocyst set [M (P_25_, P_75_),*x̄*±*s*, n(%)].

	Total	Non-PPC	PPC	*X^2^/t/Z*	*P* value
History of severe pancreatitis				29.945	<.001
No	238 (85.0)	205 (90.7)	33 (61.1)		
Yes	42 (15.0)	21 (9.3)	21 (38.9)		
Sex				2.792	.114
Male	180 (64.3)	140 (61.9)	40 (74.1)		
Female	100 (35.7)	86 (38.1)	14 (25.9)		
Hypertension				1.196	.278
No	217 (77.8)	178 (78.8)	39 (72.2)		
Yes	62 (22.2)	4 8 (21.2)	15 (27.8)		
Diabetes mellitus				12.482	<.001
No	250 (89.3)	209 (92.5)	41 (75.9)		
Yes	30 (10.7)	17 (7.5)	13 (24.1)		
History of biliary surgery				13.558	<.001
No	251 (89.6)	210 (92.9)	41 (75.9)		
Yes	29 (10.4)	16 (7.1)	13 (24.1)		
History of smoking				0.381	.597
Yes	66 (23.6)	55 (24.3)	11 (20.4)		
No	214 (76.4)	171 (75.7)	43 (79.6)		
History of drinking				1.306	.282
Yes	63 (22.5)	54 (23.9)	9 (16.7)		
No	217 (77.5)	172 (76.1)	45 (83.3)		
Abdominal cavity or pelvic effusion				14.621	<.001
No	195 (69.6)	169 (74.8)	26 (48.1)		
Yes	85 (30.4)	57 (25.2)	28 (51.9)		
Age	47.35 ± 15.502	46.82 ± 16.06	49.56 ± 12.84	−1.334	.185
RBC	4.36 ± 0.658	4.45 ± 0.64	3.94 ± 0.572	5.328	<.001
HGB	132.51 ± 22.84	136.05 ± 22.50	117.69 ± 17.91	5.588	<.001
IBIL	12.70 ± 9.522	13.49 ± 1.00	9.74 ± 6.78	2.503	.013
TP	67.15 ± 8.54	67.96 ± 8.43	63.79 ± 8.28	3.273	.001
ALB	39.05 ± 5.87	39.81 ± 5.65	35.89 ± 5.74	4.558	<.001
BMI	24.13 (22.29, 26.71)	24.49 (22.60, 27.04)	22.49 (19.92, 25.26)	−3.616	<.001
WBC	9.24 (6.24, 12.99)	10.05 (6.63, 14.07)	6.97 (5.16, 9.07)	−3.515	<.001
Percentage of neutrophils	77.85 (64.88, 84.68)	78.80 (66.78, 85.30)	66.75 (60.13, 80.43)	−3.097	.002
Neutrophil absolute value	7.22 (4.26, 10.93)	7.81 (4.67, 11.87)	4.51 (3.06, 6.91)	−3.767	<.001
PLT	194.00 (151.00, 260.00)	197.00 (149.75, 257.25)	187.50 (151.25, 295.50)	−0.093	.926
ALT	33.00 (17.00, 79.50)	37.15 (18.23, 96.90)	19.00 (11.77, 29.88)	−4.619	<.001
AST	26.00 (18.00, 50.30)	28.85 (19.15, 63.25)	17.50 (14.00, 26.75)	−4.567	<.001
ALP	87.00 (68.00, 123.00)	89.50 (68.85, 136.50)	77.00 (62.00, 93.75)	−2.240	.025
TBIL	18.15 (13.00, 26.03)	19.00 (13.65, 28.73)	13.75 (9.88, 22.10)	−3.481	<.001
DBIL	6.65 (4.40, 11.78)	6.65 (4.48, 12.33)	6.25 (3.75, 9.65)	−1.203	.229
GLO	28.20 (24.40, 31.65)	28.20 (24.28, 31.95)	28.10 (25.50, 30.98)	−0.012	.990
CA	2.20 (2.06, 2.31)	2.22 (2.06, 2.24)	2.16 (2.06, 2.25)	−2.140	.032
CREA	61.00 (48.00, 73.00)	62.00 (48.00, 74.00)	54.00 (41.00, 69.75)	−2.125	.031
BUN	4.34 (3.40, 5.60)	4.32 (3.43, 5.60)	4.34 (2.89, 5.84)	−2.340	.815
UA	309.00 (229.00, 383.20)	311.00 (233.30, 398.05)	300.80 (193.75, 337.50)	−1.633	.102
GLU	6.32 (5.18, 8.03)	6.33 (5.25, 7.90)	6.40 (4.87, 8.62)	−0.191	.848

ALB = albumin, ALP = alkaline phosphatase, ALT = alanine transferase, AST = aspartate transferase, BMI = body mass index, BUN = blood urea nitrogen, CA = blood calcium, CREA = creatinine, DBIL = direct bilirubin, GLO = glutamate oxidase, GLU = blood glucose, HGB = Hemoglobin, IBIL = indirect bilirubin, PLT = platelet, PPC = pancreatic pseudocyst, RBC = red blood cell, TBIL = total bilirubin, TP = total protein, UA = uric acid, WBC = white blood cell.

### 3.3. Multivariate analysis of the risk of secondary PPC in patients with AP

PPC secondary to AP was included as the dependent variable (No occurrence = 0; occurrence = 1). Based on the univariate analysis, the 19 significant influencing factors were included as independent variables in the logistic regression model for multivariate analysis to further screen for specific influencing factors. Variable assignments are the original income of these 15 continuous variables: RBC, HGB, IBIL, TP, ALB, BMI, WBC, percentage of neutrophils, neutrophil absolute value, ALT, AST, ALP, TBIL, CA, and CREA. History of SAP (No = 0, Yes = 1); diabetes mellitus (No = 0, Yes = 1); history of biliary surgery (No = 0, Yes = 1); and abdominal cavity or pelvic effusion (No = 0, Yes = 1). Univariate analysis revealed that a history of SAP, diabetes mellitus, history of biliary surgery, BMI, HGB, and ALB are relevant predictors of concurrent PPCs in patients with AP (Table 3).

**Table 3:** The result of the multivariate analysis.

	B	SE	*WaldX^2^*	*P* value	OR	95% CI
Constant	10.621	2.445	18.874	<.001	-	-
History of severe pancreatitis	1.560	0.508	9.432	.002	4.757	1.758–12.871
Diabetes mellitus	1.934	0.612	9.984	.002	6.919	2.084–22.967
History of biliary surgery	2.223	0.570	15.216	.000	9.232	3.022–28.203
HGB	−0.026	0.010	6.752	.009	0.974	0.955–0.994
ALB	−0.118	0.038	9.675	.002	0.888	0.825–0.957
BMI	−0.161	0.062	6.639	.010	0.851	0.753–0.962
Percentage of neutrophils	−0.028	0.014	3.781	.052	0.9772	0.945–1.000

ALB = albumin, BMI = body mass index, CI = confidence interval, HGB = hemoglobin, OR = odds ratio.

### 3.4. Nomogram development

**Figure 1.**
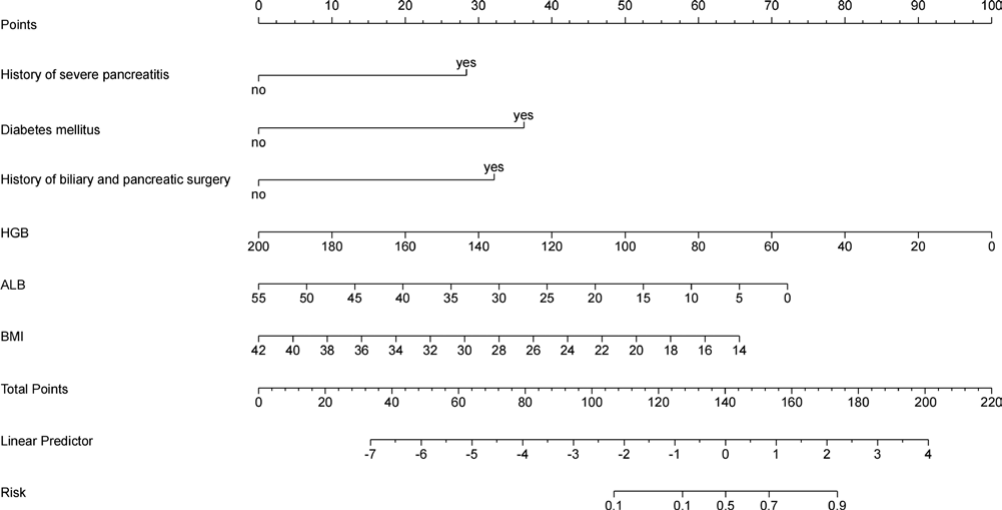
Construction and validation of the predictive nomogram for the pancreatic pseudocyst in patients with acute pancreatitis.

Multivariate logistic analysis revealed the following 6 independent risk-predictive factors for PPC: history of SAP, diabetes mellitus, history of biliary surgery, BMI, HGB, and ALB (Table 3). The nomogram was established to determine the influence of these 6 factors on PPC (Fig. 1). Diabetes mellitus had the most extended scale and significant effect on PPC. After successfully establishing the nomogram model, the R Studio software assigns different scores to the different variables. Subsequently, the total score can predict the risk of secondary PPCs according to the final summary.

### 3.5. Evaluation and validation of the dynamic nomogram

#### 3.5.1. Discrimination and calibration.

**Figure 2.**
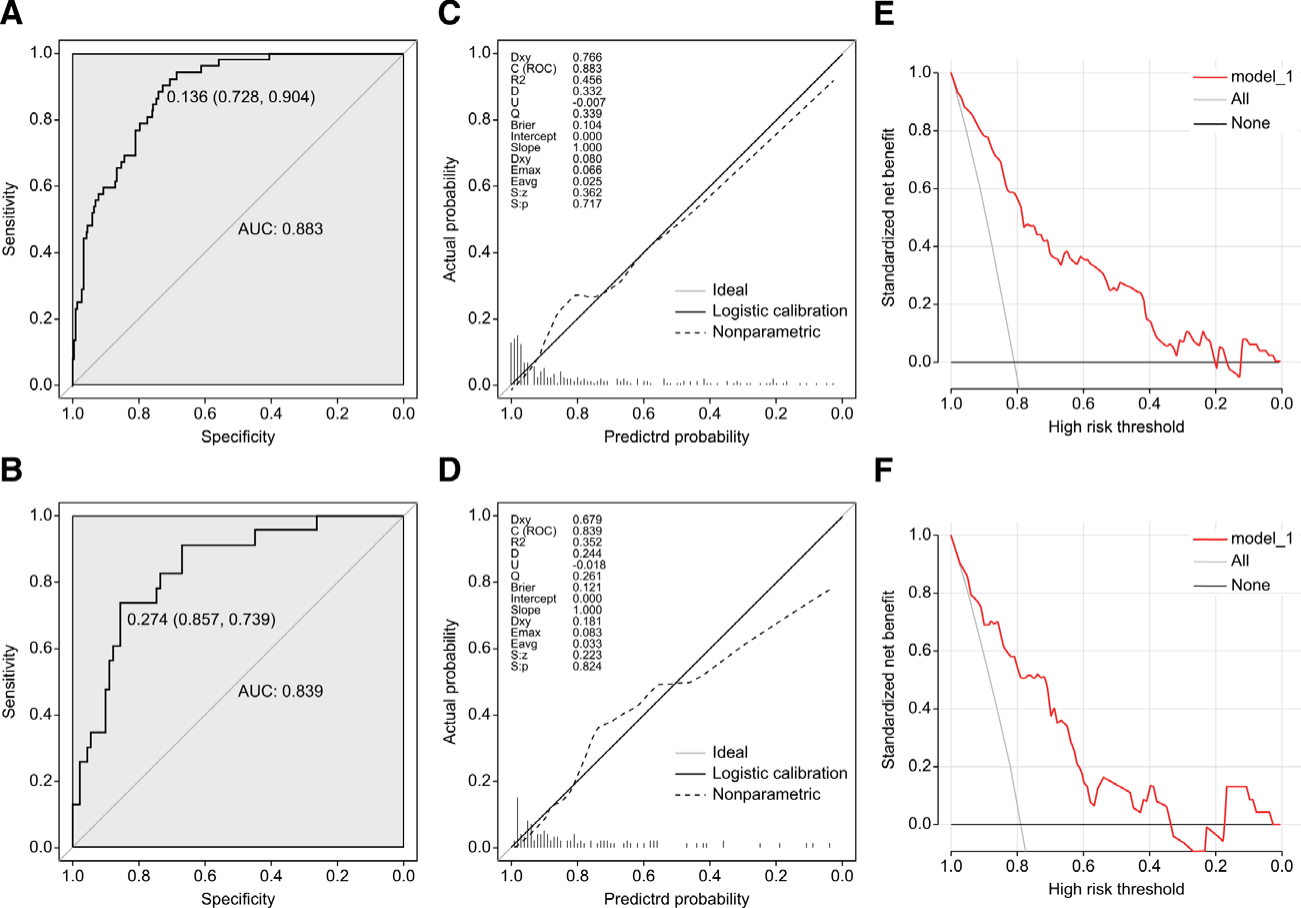
(A) Receiver operating characteristic (ROC) curves of the nomogram in the training cohort. (B) ROC curves of the nomogram in the validation cohort. (C) Calibration curve of the nomogram in the training cohort. (D) Calibration curve of the nomogram in the validation cohort. (E) Decision curve analysis in the training cohort. (F) Decision curve analysis in the validation cohort.

This study used receiver operating characteristics curves to evaluate the prediction effect of this nomogram model. Furthermore, the AUC and 95% confidence interval were calculated. The AUC and 95% confidence interval of the nomogram were 0.883 (0.839–0.927) in the training dataset (Fig. 2A) and 0.839 (0.752–0.925) in the validation set (Fig. 2B). The *P* value of the Hosmer–Lemeshow test was > .05 in the training and validation sets (*P* = .139, *P* = .544). Figure 2C and D show the calibration curves of this study. Brier scores in the training and validation groups were 0.104 and 0.121, respectively. The calibration curves and Hosmer–Lemeshow show that the predicted probability of this nomogram model is highly consistent with the actual probability, indicating that the model is well-calibrated on the training and validation sets (Figure 2C and D).

### 3.6. Decision curve analysis

The results of the nomogram model in the training and validation sets show that if the threshold probability were between 0% and 66%, using this model to predict PPC in patients with AP be more beneficial (Figure 2E and F).

## 4. Discussion

The nomogram model is a simple, accurate visual graph for predicting clinical outcomes, adverse events, or disease progressions based on multiple clinical indicators constructed in univariate and multivariate regression analyses.^[9,10]^ Recent studies on AP have searched for pathogenesis and treatment-influencing factors and have obtained considerable research progress. However, no clinical prediction study is available on AP complicated with PPCs in China and internationally. Therefore, 8 independent influencing factors were selected by univariate analysis and logistic regression by reviewing clinical medical records. Moreover, the nomogram prediction model was constructed using R Studio software. The results suggest the excellent discrimination ability of the nomogram. The calibration curve and Hosmer–Lemeshow goodness of fit test of the model suggest that the prediction deviation between the predicted and actual values of the nomogram is insignificant, indicating a good nomogram. The practicality of the results is more suitable for clinical translational application because all data in this study were derived from first-line in patients.

AP is a common acute or critical abdominal disease in clinical diagnosis and treatment. Moreover, AP is usually characterized by local inflammatory reactions in the pancreatic region. AP can be accompanied by changes in multiple organ functions.^[11–13]^ PPC is a common clinical complication in patients with AP. It usually occurs during the clinical recovery phase after a patient develops AP.^[14]^ A previous study demonstrated that the incidence of concurrent PPCs in patients with AP is approximately 6.0% to 19.0%.^[13]^ However, the incidence of PPC in this study was 19.75%. This result is slightly higher than that observed in previous studies.^[15]^ This discrepancy may be because patients with pancreatitis in our hospital were mostly SAP. The incidence of PPCs in this study was relatively high because it is the most common complication in SAP. PPCs can significantly prolong the length of hospital stay and follow-up time and increase the economic burden on patients and expenditure of health insurance funds. Therefore, establishing a clinical risk prediction model of PPC for high-risk pancreatitis patients with preliminary screening and pre-intervention to achieve strict follow-up, early detection, diagnosis, and treatment, to reduce the incidence of complications, mortality rate, and average hospital stay, and to save the expenditure of public medical resources consumption has important clinical significance.^[16]^

This study revealed that SAP history, diabetes mellitus, history of biliary surgery, BMI, HGB, and ALB were the main influencing factors of pancreatitis and PPC. There are specific reasons for analysis. For example, SAP can lead to extravasation of pancreatic fluid, tissue liquefaction necrosis products, and inflammatory exudate in the peripancreas or pancreatic aggregation that cannot be absorbed to form fiber or granulation tissue package, leading to pseudocyst formation. The possibility of concurrent pseudocyst is lower in patients with mild pancreatitis than that in patients with SAP because the inflammatory exudate of these patients is relatively mild.^[16,17]^ This study revealed that SAP history is also one of the main influencing factors of AP complicated with PPCs, consistent with the results of existing international research.^[18,19]^ This may be because recurrent AP aggravates the burden on the pancreas, causing some remodeling of the tissue anatomy and structure. This increases the aggregation of effusions, such as pancreatic fluid, inducing pseudocysts.^[18]^ Our results show that the history of biliary surgery is an important factor with PPCs, and the risk is much higher than that of the other influencing factors because the operation of cholanopancreatic changes in the structure of the gallbladder and pancreas forms a scar in the bile and pancreatic ducts, which further changes their structures, increasing the risk of PPC. Diabetes mellitus is also an independent factor affecting PPC, consistent with previous studies.^[20–23]^ This is because poor glycemic control can lead to microvascular atherosclerotic lesions and cause vascular stenosis, affecting the tissue perfusion function and microcirculation. Furthermore, AP can lead to pancreatic edema, which can hinder insulin secretion and affect the patient’s blood sugar level.^[24]^ Higher blood sugar can lead to various complications in the body or a systemic inflammatory response in patients. SIRS will lead to gluconeogenesis, increasing the blood sugar value and burden on the pancreas, leading to the progressive aggravation of inflammatory reactions. The aggravation of pancreatic inflammatory response can increase the aggregation of inflammatory secretions, creating objective environmental conditions for pseudocyst formation, thereby increasing the incidence of pseudocysts.^[25]^ BMI is closely associated with pancreatitis and concurrent PPCs. In the diagnosis and treatment guidelines of AP, the BMI value is included in the clinical indicators to assess the degree of pancreatitis disease.^[2]^ This conclusion is consistent with our results, and previous studies have shown that the severity and prognosis of AP are closely related to the BMI value.^[21,26,27]^ Meanwhile, more studies have shown that after the BMI value reaches obesity, the severity of BMI and AP, probability of recurrence, and risk probability of complications gradually increase.^[28–31]^ Obese and overweight patients have more fat accumulation in organs and thus, have excessive accumulation of fat around the pancreas. Therefore, pancreatic bleeding, effusion package aggregation, and saponification reactions in AP provide an adequate environment and conditions. Similarly, inflammatory and pancreatic fluid exudates will lead to fat decomposition, producing numerous free fatty acids and directly or indirectly cause pancreatic damage, aggravating the condition^[19]^ and resulting in a surge of complications. The response of HGB to the patient is the patient’s hematopoiesis, and AP is a wasting disease. Therefore, AP may lead to anemia and other symptoms, affecting the prognosis and recovery of patients, resulting in a vicious circle of the body, which may induce various complications. This study discovered that serum ALB is one of the main influencing factors of PPC secondary to pancreatitis, consistent with previous studies.^[32,33]^ AP can put the body under stress, increasing the body’s capillary permeability and ALB loss. Furthermore, increased pancreatic inflammatory exudate and fluid accumulation of space consume serum ALB, causing serum ALB progressive decline, leading to hypoproteinemia. Hypoproteinemia aggravates the increase and aggregation of the abdominal cavity, peripancreatic exudates, and the decrease of body absorption, thus creating conditions for the formation of encapsulating effusion and promoting pseudocyst formation.^[16,34]^

This study has some limitations. This is a single-center, retrospective study; hence, the sample source and size are insufficient, and the effect of regional characteristics on the disease cannot be excluded. Due to rigor, this study excluded medical records with irregular record writing and imperfect data in the data collection stage, introducing some publication bias in the results. This study only conducted an internal verification of the constructed nomogram prediction model, making the external validity of the study results unknown. Therefore, follow-up research should conduct multi-center large sample research to verify these findings and build a standard prediction tool suitable for global promotion.

## 5. Conclusion

This study determined whether the experience of severe illness, recurrence, diabetes, history of biliary surgery, BMI, neutrophil percentage, hemoglobin, and ALB are the main influencing factors of PPC. Furthermore, this study constructed a clinical predictive nomogram model. The validation results of the nomogram model have excellent predictive accuracy and high clinical predictive guiding value.

## Author contributions

**Conceptualization:** Shanbing Hou.

**Data curation:** Shanbing Hou, Lanlan Yang.

**Formal analysis:** Shanbing Hou, Ming Dou.

**Funding acquisition:** Ming Dou.

**Investigation:** Shanbing Hou, Senlin Wang, Yuetong You, Lanlan Yang.

**Methodology:** Shanbing Hou, Yuetong You, Lanlan Yang, Ying Zhang.

**Project administration:** Shanbing Hou, Yuetong You, Ming Dou, Ying Zhang.

**Resources:** Shanbing Hou, Yuetong You, Ming Dou, Ying Zhang.

**Software:** Shanbing Hou, Ying Zhang.

**Validation:** Shanbing Hou.

**Writing – original draft:** Shanbing Hou, Senlin Wang.

**Writing – review & editing:** Shanbing Hou.
